# A Multimodal In-Ear Audio and Physiological Dataset for Swallowing and Non-Verbal Event Classification

**DOI:** 10.3390/s26072019

**Published:** 2026-03-24

**Authors:** Elyes Ben Cheikh, Yassine Mrabet, Catherine Laporte, Rachel E. Bouserhal

**Affiliations:** 1 The Research in Hearing Health and Assistive Devices (RHAD) Lab, École de technologie supérieure (ÉTS), Montreal, QC H3C 1K3, Canada; elyes.ben-cheikh.1@ens.etsmtl.ca (E.B.C.); yassine.mrabet.1@ens.etsmtl.ca (Y.M.); 2 Laboratoire de Traitement de l’Information en Santé (LATIS), École de technologie supérieure (ÉTS), Montreal, QC H3C 1K3, Canada; catherine.laporte@etsmtl.ca

**Keywords:** multimodal dataset, in-ear microphone, non-verbal events, swallowing classification

## Abstract

Swallowing is a critical marker of neurological and emotional health. The ability to monitor it continuously and non-invasively, especially through smart ear-worn devices, holds significant promise for clinical applications. Despite this potential, no public audio datasets currently support reliable swallowing sound detection. Existing datasets focus primarily on speech and breathing, offering limited coverage and lacking detailed annotations for swallowing events. To address this gap, we introduce an in-ear audio dataset specifically designed to capture a wide range of verbal and non-verbal sounds. It includes comprehensive labeling focused on swallowing. The dataset was collected from 34 healthy adults (14 females and 20 males) between the ages of 20 and 29. Each participant performed a series of predefined tasks involving both non-verbal and verbal events. Non-verbal tasks included swallowing, clicking, forceful blinking, touching the scalp, and physical movements such as squatting or walking in place. Verbal tasks consisted of speaking (e.g., describing an image). Recordings were conducted in both quiet and noisy environments to better reflect real-world conditions. Data were captured using a combination of in-/outer-ear microphones, a chest belt to record electrocardiogram (ECG), respiration and acceleration signals, and an ultrasound probe to track tongue movement, which served as a reference for swallowing annotation. All signals were precisely synchronized. To ensure high data quality, the recordings were reviewed using both algorithmic analysis and manual inspection. Swallowing events were identified based on ultrasound signals and validated by an expert to guarantee accurate labeling. As a proof of concept that in-ear audio supports swallow classification, we fine-tune a fully connected neural network on YAMNet embeddings plus zero-crossing rate (ZCR) features. Across the completed folds, the model reaches an F1 score of 0.875 ± 0.013.

## 1. Introduction

Swallowing is a vital physiological function that ensures the safe transport of food from the oral cavity to the esophagus while protecting the airway [1]. A disruption of this mechanism can cause dysphagia, which affects many adults in relation to various neurological disorders [2]. In Parkinson’s disease, sialorrhea, or excessive salivation, is mainly due to a lower frequency of swallowing rather than increased saliva production [3]. This impaired swallowing in Parkinson’s disease patients leads to social embarrassment and promotes saliva inhalation into the lungs, posing a risk of respiratory complications [3]. Similarly, patients with motor neuron disease exhibit a reduction in swallowing ability compared to healthy subjects [4]. Beyond neurological disorders, the frequency of spontaneous swallowing is also modulated by emotional state or stress. Studies in healthy volunteers have shown that stress or anxiety significantly increases the rate of swallowing compared to a relaxed state [5]. Moreover, sensory or cognitive factors such as hunger, attention, or exposure to food-related stimuli like pleasant smells or visual presentation of food can also influence swallowing activity [6,7]. Thus, swallowing is not only a marker of neurological or physiological health but also reflects behavioral, sensory, and emotional states. Therefore, monitoring swallowing has a dual clinical value: it allows for detecting and tracking the progression of swallowing disorders, while also providing insights into the patient’s autonomic and behavioral states (stress, pain or hunger) through the frequency and context of spontaneous swallowing.

Physiological factors related to the swallowing act itself also influence the acoustic characteristics of swallowing sounds. In particular, variations in bolus properties and subject age have been shown to significantly affect the spectral and temporal features recorded from both ear and cervical sensors [8]. Such variability introduces additional challenges for automatic swallowing detection systems, as swallowing signals may differ substantially both between subjects and across repeated swallows within the same individual.

Given the clinical relevance of swallowing, researchers are exploring technological approaches to continuously monitor this function in daily life.

Multimodal measurements are increasingly viewed as essential for reliable physiological monitoring. Rather than relying on a single channel, recent systems deliberately combine complementary modalities that capture different aspects of swallowing physiology. Shin et al., for example, integrated two surface electromyography (EMG) electrodes and a microphone on a kirigami-structured neck wearable, achieving about 89.5% accuracy in swallowing and silent aspiration detection with clinically competitive performance [9]. Similarly, Song et al. introduced a soft, skin-attachable throat vibration sensor and demonstrated deep-learning-based classification of multiple throat events (e.g., cough, speech, swallowing), reporting an accuracy of approximately 96% [10]. Beyond performance gains, these studies highlight a key motivation for multimodal sensing: combining heterogeneous signals reduces ambiguity, improves robustness to noise and motion artifacts, and mitigates inter-subject variability by allowing one modality to compensate when another becomes unreliable. However, despite the clear advantages of multimodal fusion, progress is constrained by the limited availability of high-quality, synchronized multimodal datasets with reliable reference annotations.

Recent work on automatic swallow detection has primarily relied on neck-based sensing, including cervical auscultation microphones, accelerometers, and surface EMG. Using these modalities, Gravellier et al. reported multi-event pharyngolaryngeal activity detection in ecological conditions with a depthwise CNN–LSTM model (F1 = 0.88 for swallowing; 42 participants) [11]. Kimura et al. achieved F1 ≈ 0.92 and 95.2% accuracy on videofluoroscopic swallowing study (VFSS) synchronized recordings using mel-frequency cepstral coefficient (MFCC) features and ensemble classifiers [12]. More recently, So et al. extended cervical auscultation approaches to pediatric and neonatal cohorts using transfer learning, reporting 91% accuracy [13]. While these results demonstrate the effectiveness of neck-based pipelines, the required sensor placement may be obtrusive and therefore less suited to comfortable, long-term monitoring in daily life.

The recent emergence of smart wearable auditory devices, commonly known as hearables, offers new opportunities for the non-invasive monitoring of vital signs and physiological signals. Recent developments in hearable hardware platforms further highlight the ear as a promising location for physiological sensing. For example, the open-source OpenEarable 2.0 platform integrates multiple sensors within an ear-worn device, enabling multimodal physiological monitoring [14]. Hearables can operate with or without an acoustic seal. Open-fit earbuds admit external sound and produce little to no occlusion effect, whereas in-ear microphone (IEM) devices form a tight seal in the ear canal when properly placed. Compared to conventional throat or neck placements, a sealed ear canal benefits from the occlusion effect. This seal plays a crucial role by reducing external noise. This effect results in the amplification of internally generated sounds such as breathing, swallowing, or muscle movements through bone and tissue conduction. As a result, vibrations are amplified within the sealed canal, allowing the IEM to capture subtle non-verbal physiological signals [15].

Several studies have demonstrated the feasibility of measuring respiratory rate and heart rate using a microphone placed in the ear, even in noisy environments [16,17]. For example, audio recordings in the ear canal allow the extraction of breathing and heartbeat sounds with acceptable accuracy after denoising, even at ambient noise levels up to 110 dB [17]. Similarly, in-ear recordings of respiratory sounds have been used to classify respiratory phases (inspiration vs. expiration) and to distinguish nasal from oral breathing [18]. These findings confirm the potential of IEMs to capture physiological signals [19].

More recently, this approach has been extended to swallowing: sensors embedded in earpieces have shown the ability to detect certain movements associated with swallowing. In particular, Yoshimoto et al. [20] showed that an earphone type optical sensor, consisting of an in-ear IR LED and phototransistor that convert changes in reflected light from the ear canal motion into electrical signals, can track soft-palate motion during swallowing when compared with simultaneous videofluoroscopy (the clinical gold standard). In another study, intra-aural acoustic recordings synchronized with videofluoroscopy demonstrated that ear-canal microphones can capture swallowing-related sound patterns that are temporally aligned with physiological swallowing events [21]. Automated segmentation of swallowing sounds has also been validated against videofluoroscopic swallowing studies to estimate clinically relevant parameters such as pharyngeal clearance time [22]. In-ear sensing has also been explored for detecting mastication. Papapanagiotou et al. [23] developed a chewing detection system combining audio, photoplethysmography (PPG), and accelerometry, demonstrating that multimodal fusion improves the automatic recognition of eating activity.

These advancements highlight how hearables, beyond their audio capabilities, are emerging as health monitoring platforms capable of continuously and discreetly tracking respiration, pulse, chewing, or swallowing.

Despite these promising developments, there are very few publicly available datasets suitable for training and validating automatic swallowing detection algorithms. As summarized in Table 1, existing ear-worn and in-ear microphone (IEM) datasets can be broadly divided into speech-oriented, respiration-focused, or activity-based corpora. Although valuable for their respective objectives, these resources present important limitations when considered for precise swallowing analysis. Speech-oriented datasets were primarily developed for voice acquisition rather than physiological event detection.

Existing datasets can be grouped into speech, respiration, and activity-oriented corpora, each addressing different objectives but remaining limited for swallowing validation. SpEAR [24] provides synchronized in-ear and outer-ear microphone recordings from 25 participants, yet focuses exclusively on speech without physiological or swallowing annotations. VibraVox [25] extends this approach to a large-scale multimodal corpus (188 participants) including IEMs, bone conduction sensors, and a laryngophone, but does not provide event-level or imaging-supported swallowing labels. On the respiration side, iBad [15] combines in-ear audio with ECG and respiratory belt signals to study breathing dynamics, although swallowing events are neither specifically annotated nor imaging-validated. Similarly, RespEar [26] links IEM recordings to respiratory references but concentrates on respiration rate estimation rather than event detection. Finally, activity-oriented datasets such as OESense [27] incorporate chewing and motion events with partial video validation, yet lack complementary physiological biosignals and imaging-supported swallowing annotations. As a result, none of these publicly available datasets integrates synchronized in-ear audio, multimodal physiological measurements, and imaging-validated swallow annotations with precise temporal boundaries for benchmarking automatic swallowing detection.

In this context, we introduce a multimodal dataset covering varied verbal and non-verbal events, with comprehensive annotations and a focus on swallowing. Synchronized in-/outer-ear audio, respiration, ECG, acceleration and lingual ultrasound were recorded from healthy adult participants performing spontaneous (natural rate) or prompt swallows under three opposing acoustic conditions: one quiet environment and two noisy ones. Finally, we present a proof of concept demonstrating the relevance of this multimodal dataset for swallowing analysis through a binary swallow classification task.

## 2. Methods

In this study, data collection and analysis were conducted following ethical approval from the CER, the IRB of the École de technologie supérieure (H20221006). All procedures were carried out according to the relevant guidelines and regulations.

### 2.1. Data Collection

Participants were recruited through email invitations and word-of-mouth referrals. A total of 34 individuals (14 females and 20 males), aged between 20 and 29, were enrolled in the study. Before data collection, all participants underwent an otoscopic examination to assess the condition of the outer ear and the eardrum. This evaluation ensured the absence of foreign objects, excessive cerumen, or signs of infection or inflammation. Only participants deemed healthy and free of ear-related conditions were included.

#### 2.1.1. Sensors and Instrumentation

Acoustic data were collected inside a double-wall audiometric booth (Eckel Noise Control Technologies, Morrisburg, ON, Canada) using a Roland OCTA-CAPTURE USB audio interface (Roland Corp., Hamamatsu, Japan) operated via MATLAB 2022 (MathWorks, Natick, MA, USA). Recordings were made on four channels at a sampling rate of 48 kHz with 16-bit resolution, capturing signals simultaneously from the left and right ears using custom earpieces developed by the ÉTS-EERS Industrial Research Chair in In-Ear Technologies. Each earpiece was equipped with two microphones: one positioned inside the ear canal and the other outside the ear. Both microphones were Knowles FG45-32491-000 electret condenser capsules (Knowles Electronics LLC, Itasca, IL, USA). The nominal sensitivity is −58 dB re 1 V/Pa at 1 kHz, which corresponds to approximately 1.26 mV/Pa. The intra-aural microphone configuration is based on previously validated in-ear sensing technology, shown to reliably capture physiological acoustic signals [24]. Background noise was delivered through four loudspeakers placed within the booth.

Physiological signals were captured using the BioHarness™ 3.0 wearable chest belt (Zephyr Technology Corporation, Annapolis, MD, USA), a clinically and experimentally validated system for ambulatory monitoring of multiple biosignals, with demonstrated accuracy and reliability for respiration and cardiac measurements across rest and exercise conditions [28,29]. The device, equipped with a chest strap containing dry electrodes and a sensor module, recorded a single-lead ECG at 250 Hz, respiration rate through a pressure sensor at 25 Hz, and triaxial accelerometry at 100 Hz. Prior to each session, the device was configured in logging mode using the Zephyr Configuration Tool.

Based on evidence showing that ultrasound of the submental region allows reliable visualization of swallowing-related movements [30], ultrasound imaging of the tongue through the submental region was performed using a pocket-sized MicrUs USB-powered system (Telemed, Vilnius, Lithuania) equipped with an MC4-2R20S-3 probe, (4 MHz center frequency; 90 mm depth; 66 dB dynamic range). The system was operated with EchoWave 2 software (v4.4) on a laptop with an Intel Core i7 processor, 16 GB of RAM and a 64-bit operating system. Ultrasound frames were captured at 30 frames per second, while OBS Studio (v30.2) simultaneously recorded the on-screen ultrasound video along with an audio monitoring channel sampled at 44.1 kHz. Pocket-sized ultrasound systems have previously demonstrated validity and reliability for swallowing assessment, supporting their suitability for non-invasive and repeatable evaluation of swallowing biomechanics [31].

**Figure 1:**
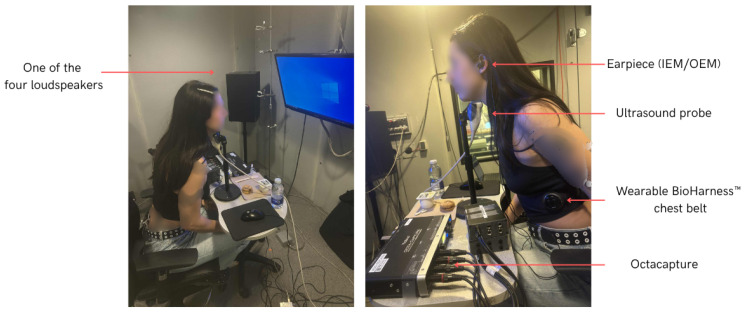
An example showing the setup and the placement of the different sensors during the recording.

An example of the setup, including a participant equipped with the sensors, is shown in Figure 1.

#### 2.1.2. Procedure

**Figure 2:**
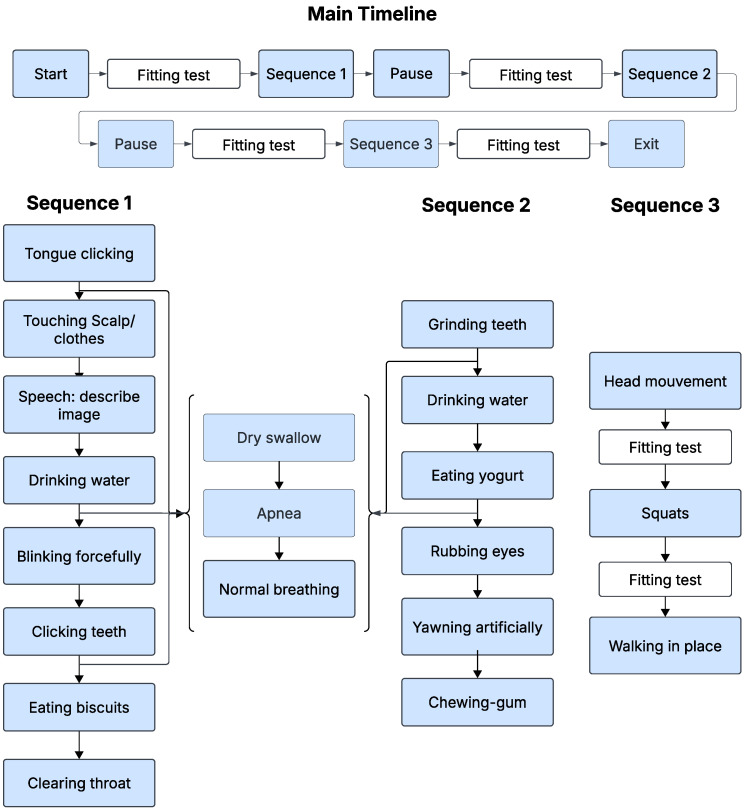
Sequence diagram of the experimental protocol.

The protocol was repeated three times under different auditory conditions: quiet, babble and factory noise with identical procedures per session, as illustrated in Figure 2. Longitudinal recordings were not included in this study. Prior physiological studies report no significant test–retest differences in swallowing timing measures across sessions in healthy individuals [32]. Each session began with a fitting test to ensure proper placement: a good acoustical seal and, therefore, optimal signal quality. This fitting test was repeated as many times as necessary until the quality criteria defined in Section 2.1.3 were met, ensuring reliable data acquisition throughout the session. After the initial fitting validation, the participants proceeded to Sequence 1, as shown in Figure 2, which included various tasks such as tongue clicking, blinking, scalp touching, drinking water, clicking teeth, eating biscuits or clearing the throat. Upon completion of this sequence, participants were given a pause period before undergoing a new fitting test, again ensuring correct positioning and signal integrity. This was followed by Sequence 2, which involved actions such as teeth grinding, drinking water, eating yogurt, rubbing the eyes, artificially yawning, and chewing gum. After a second pause, Sequence 3 follows, where participants performed head movements, squats, and walking in place, interspersed with fit tests between activities to confirm ongoing fitting adequacy, particularly after physical activities that could affect device positioning. In this sequence, no ultrasound was used. Sequence 3 was included to capture motion-related artifacts and physiological variability in heart rate and respiration. Alternating between sequences 1 and 2, participants also carried out specific inter-sequence events including dry swallowing, a voluntary 10 s apnea, and a return to regular breathing. At the end of each session, a final fitting test was performed prior to concluding the recording. This structured approach ensured that the device fitting was carefully controlled and maintained at every step of the experiment. Before each activity, participants pressed the Start button in the MATLAB interface to mark the onset. Immediately after completing the activity, they pressed an End button to mark the offset. Both markers were generated and time-stamped by the same MATLAB process that controlled signal acquisition, sharing the acquisition clock. This provided an initial coarse, time-synchronized annotation of the recordings, including the activity class.

#### 2.1.3. Acoustic Seal Validation

To verify an adequate acoustic seal, pink noise at 85 dBA was played through four loudspeakers placed in the audiometric room. The custom earpiece fit was considered acceptable if the attenuation between the OEM and the IEM signals was at least 8 dB at 250 Hz [33]. The foam tips of the earpieces were adjusted as needed, and different sizes were used depending on each participant’s ear canal dimensions to ensure proper insertion and sealing.

#### 2.1.4. Multimodal Synchronization

Data were acquired using three different sensing systems: custom earpieces for in-ear audio signals, a chest belt for physiological measurements including ECG, respiration, and acceleration, and an ultrasound system for imaging tongue movement. As these modalities operated independently, a two-step synchronization protocol was required to align the recordings.

##### First Synchronization Step: Earpiece and Ultrasound System

**Figure 3:**
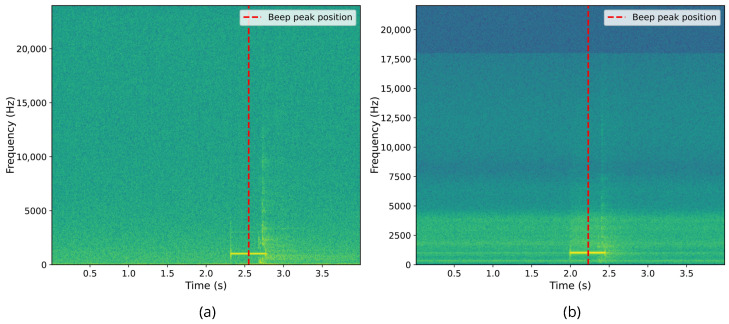
First synchronization marker alignment. (**a**) Spectrogram of the beep recorded from right ear IEM microphone. (**b**) Spectrogram of the beep recorded from the external microphone, synchronized with ultrasound via OBS.

The initial synchronization step aligned the earpiece and ultrasound data streams using an audio cue. Specifically, at the beginning of the recording protocol, a 500 ms beep was played, which was simultaneously recorded by the external microphone (capturing the ultrasound display) and by the earpiece’s microphone channels. This beep, recorded by both the external microphone and the OEM, was subsequently identified in the recordings through autocorrelation analysis between the external microphone signal and the ultrasound video audio track, as well as between the OEM signal and the earpiece audio recording. We then shifted the delayed signal by the estimated offset to align the beep onsets. Given the audio sampling rates of 48 kHz and 44.1 kHz, the temporal resolution of this alignment is limited to one sample period, corresponding to a synchronization uncertainty of approximately ±0.02 ms. Figure 3 illustrates the detection of the synchronization beep in both data streams.

##### Second Synchronization Step: Earpiece and Chest Belt via Respiration

**Figure 4:**
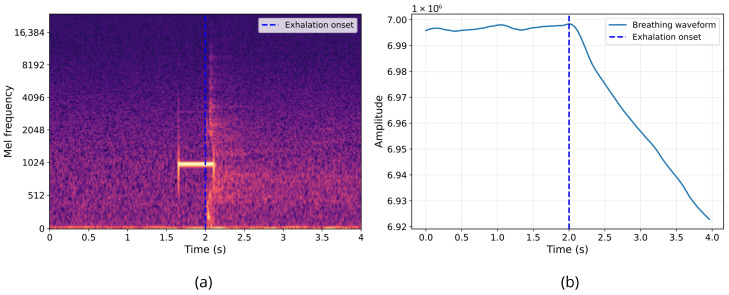
Second synchronization marker alignment. (**a**) Spectrogram of the exhalation sound recorded by right ear IEM microphone. (**b**) Breathing waveform recorded by BioHarness 3.0 device.

The second synchronization step aligned the audio from the earpiece and the data from the chest belt using the respiration signal as shown in Figure 4. Participants were instructed to hold their breath for 10 s at the start of the protocol, followed by an immediate exhalation after the synchronization beep. This maneuver allowed the identification of the exhalation event in both the OEM signals and the respiration signal. The respiration signal exhibited a plateau corresponding to breath-holding. This was followed by a marked change indicating the onset of exhalation, characterized by a progressive decrease in lung volume.

Simultaneously, the sound of exhalation was visible in the earpiece audio spectrogram. Since the earpiece and ultrasound data streams had already been synchronized in the first step, aligning the earpiece and chest belt signals via respiration allowed for the synchronization of all three data streams. Since the respiration signal was sampled at 25 Hz, the synchronization uncertainty is limited to one sample period, corresponding to ±40 ms. This represents the maximum cross-modal synchronization error.

#### 2.1.5. Labeling

Labeling targeted four types of swallow: saliva, water, yogurt, and biscuits. Within the start and end markers defined in Section 2.1.2, we reviewed each 30 s activity window using synchronized ultrasound video and in-ear audio. Ultrasound recordings were examined frame by frame to identify candidate events that show the characteristic propulsion/retraction pattern of the tongue associated with swallowing, which were then confirmed when a temporally aligned swallow sound was observed in the IEM recording. Ultrasound was recorded at 30 fps (33 ms per frame); to avoid onset and offset ambiguity at the frame level, each swallow label was padded by one frame (±33 ms), so the entire event is reliably included in the annotated segment. For each confirmed event, onset and offset times were annotated. All annotations were performed by a single rater. A subset of labels was independently checked by an expert.

### 2.2. Swallowing Binary Classification

The study initially enrolled 34 healthy young adults. Prior to analysis, several participants were excluded because of missing or unreliable swallow annotations due to incomplete ultrasound labeling. The final dataset used for classification included 26 participants and 8202 annotated acoustic segments.

For binary swallowing classification, any segment containing a swallow regardless of bolus type (saliva, water, yogurt, or biscuit during mastication) was assigned to the positive class (label = 1). All remaining segments were assigned to the negative class (label = 0), which contains quiet respiration or silence, saliva mix sounds, and physiological noise.

#### 2.2.1. Preprocessing

All analyses used in-ear recordings captured at 48 kHz. Recordings were normalized and split into fixed 4 s windows via zero-padding or truncation as needed to match the maximum swallow duration observed and to have a consistent input size. Each segment was resampled to 8kHz to match the effective bandwidth of in-ear recordings imposed by the occlusion effect [34] (Nyquist preserves content up to 4kHz).

#### 2.2.2. Classification

**Figure 5:**
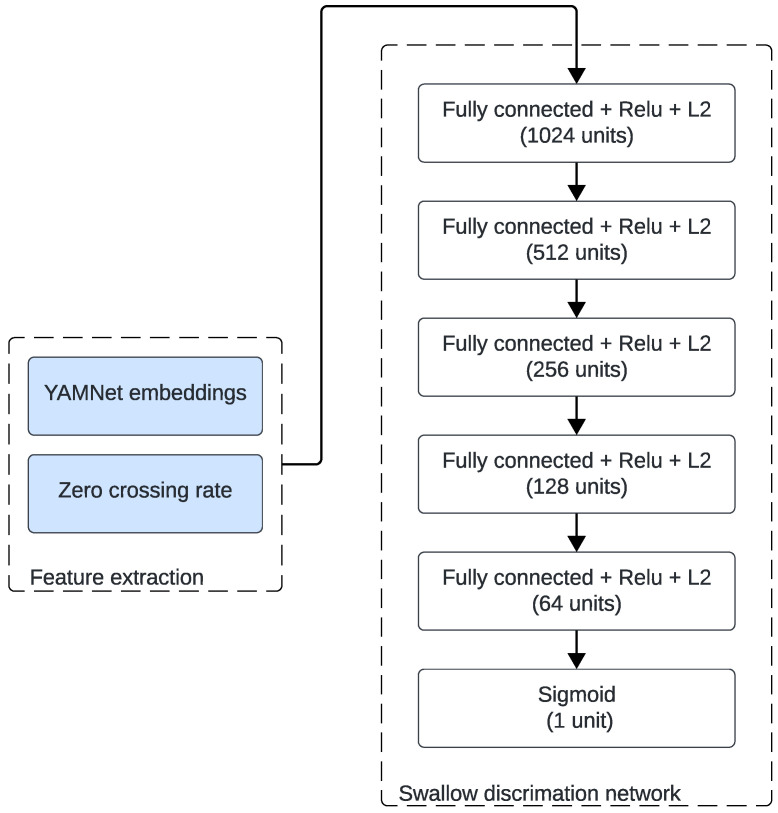
Fully connected network for swallow classification.

The choice of YAMNet was motivated by its strong generalization ability for environmental and non-speech acoustic events. This pre-trained model provides robust frame-level embeddings that capture both spectral and temporal features relevant to short impulsive body sounds such as swallows. Moreover, previous work by So et al. [13] demonstrated that YAMNet embeddings used as a feature extraction stage achieved high performance for swallow sound detection, confirming its suitability for this task. We therefore adopted the same backbone configuration and adapted it to our specific frequency range. Figure 5 shows the employed architecture. Each preprocessed segment was fed to the YAMNet backbone adapted for low-rate audio (8 kHz). YAMNet produces a sequence of T×1024 frame-level embeddings, where *T* is the number of frames obtained by sliding YAMNet’s 0.96s analysis window with 50% overlap, as used during its AudioSet pretraining [35]. We then summarized these embeddings by computing their temporal mean and standard deviation, yielding two 1024-dimensional summary vectors. In parallel, we computed the zero-crossing rate (ZCR) on the 8 kHz waveform using a 0.96 s analysis window with 50% overlap, and summarized ZCR by its mean and standard deviation. As described by [13], we concatenated both features to form a fixed-length 2050-dimensional descriptor:

$$x=\left[\mu_{e},\;\sigma_{e},\;\mu_{\mathrm{ZCR}},\;\sigma_{\mathrm{ZCR}}\right]\in R^{2050}.$$

The dataset was imbalanced, with 60% non-swallow and 40% swallow segments. Within the swallow class, 46% were water, 34% saliva, 15% yogurt, and 5% biscuit. We addressed class imbalance only during training using dynamic batch balancing: each mini-batch was constructed to contain exactly half swallow and half non-swallow samples, with the swallow class further stratified so that saliva, water, yogurt and biscuit each represented 25%. Importantly, the validation and test sets were left untouched, preserving their natural class distributions.

In the transfer learning approach used in this study, the softmax layer of YAMNet was then replaced with a fully connected neural network (FCNN). The FCNN comprised five hidden layers, each followed by batch normalization, ReLU activation, and dropout regularization that were chosen empirically, with values ranging from 0.3 to 0.4. The output layer consisted of a single sigmoid unit that estimated the probability of a swallow event.

Before training, all features were standardized using statistics from the training set only to avoid information leakage. Optimization used the AdamW optimizer with a learning rate of $1\times 10^{-3}$ and a weight decay of $1\times 10^{-5}$. Training was regularized with early stopping based on validation loss, saving the best weights, and a Reduce on Plateau schedule that halved the learning rate whenever validation performance did not show further improvement. Finally, to ensure a subject-independent evaluation, we adopted a 5-fold cross-validation where folds were defined by participant IDs. In each fold, approximately 80% of participants (21 out of 26) were allocated to the training set and 20% (5 out of 26) to the test set. Within the training set, 15% of participants were further reserved for validation.

## 3. Results

### 3.1. Dataset Overview

This section presents representative excerpts from the collected dataset to illustrate the nature and quality of the acquired signals.

**Figure 6:**
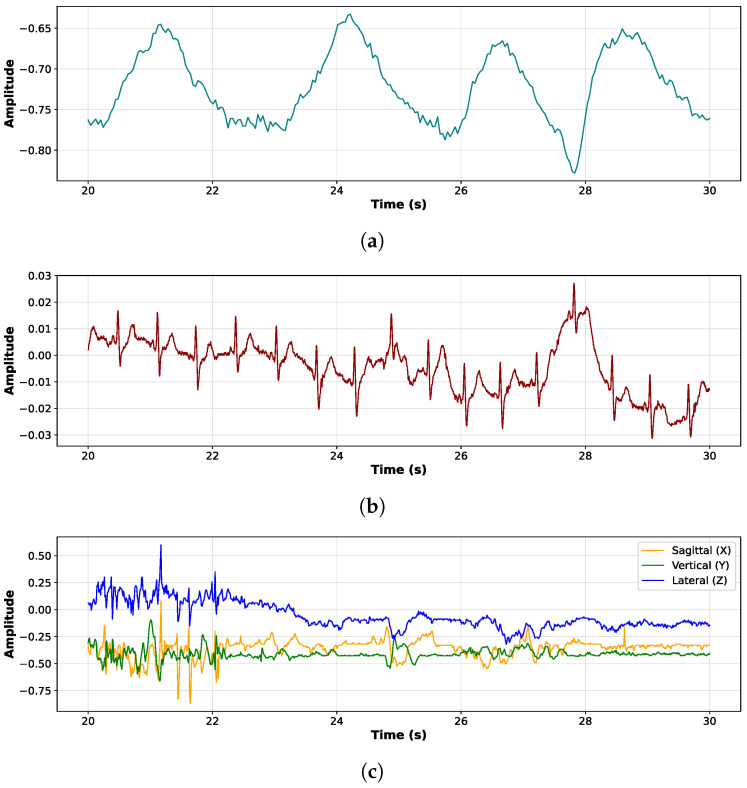
Three synchronized physiological signals recorded with the Zephyr chest belt, over a 10 s window for participant 27: (**a**) breathing, (**b**) ECG, and (**c**) accelerometer signals.

Figure 6 presents three synchronized physiological signals recorded over a 10 s window. The top panel, Figure 6a, shows the respiratory waveform, characterized by smooth, periodic oscillations corresponding to inhalation and exhalation cycles. This signal reflects a regular breathing pattern, captured via a chest belt sensor. The middle panel, Figure 6b, displays the ECG signal, where sharp and repeated peaks representing QRS waves indicate a stable heart rhythm. Slight amplitude modulations may suggest movement artifacts. The bottom panel, Figure 6c, shows triaxial accelerometer data along the sagittal (X), vertical (Y), and lateral (Z) axes. Variations in these signals reflect subtle movements of the body or head, potentially related to breathing, swallowing, or postural adjustments.

**Figure 7:**
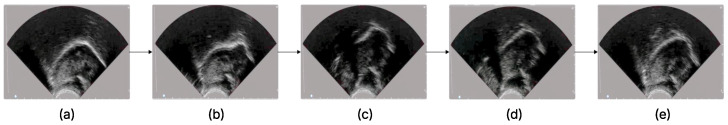
Ultrasound imaging of tongue during swallowing stages. (**a**) Resting position of the tongue prior to swallowing. (**b**) Tongue curving upward and backward, initiating bolus propulsion toward the pharynx. (**c**) Tongue retraction and floor-of-mouth muscle engagement as the bolus advances posteriorly. (**d**) Continued posterior progression of the bolus. (**e**) Repositioning of the tongue following swallowing.

As shown in Figure 7, the different panels illustrate several stages of the swallowing process captured by submental ultrasound imaging.

The observed tongue motion pattern is distinctive of swallowing; while tongue movements also occur during speech, their ultrasound characteristics differ and speech events are identifiable in the microphone recordings.

**Figure 8:**
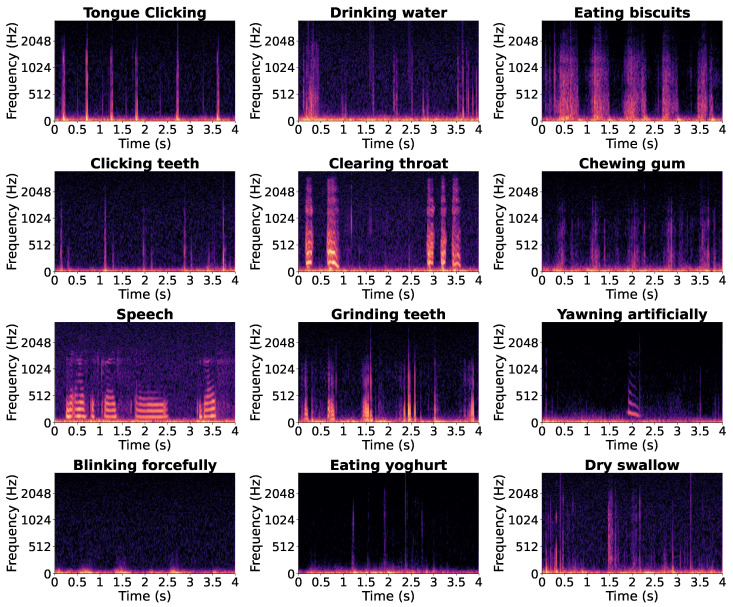
Twelve spectrograms of 4 s in-ear audio segments recorded during different verbal and non-verbal events.

Figure 8 shows twelve representative 4 s in-ear audio spectrograms recorded during different verbal and non-verbal events, illustrating the acoustic diversity captured in the dataset.

**Figure 9:**
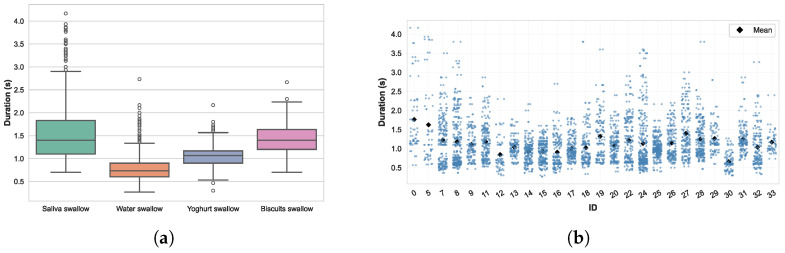
Analysis of swallowing durations across textures and intra-individual variability. (**a**) Swallowing duration by texture. (**b**) Intra-individual variability in swallowing duration.

The swallow dataset consists of 1632 dry saliva swallows, 2208 water swallows, 740 yogurt swallows, and 228 biscuit swallows, highlighting an imbalance between textures. The distribution of swallowing durations by texture, as shown in Figure 9a, shows clear differences. A linear mixed-effects model with texture as a fixed effect and a random intercept per participant confirmed a significant influence of texture on swallowing duration (p<0.001). On average, saliva swallows were the longest (M=1.55±0.59s), followed by yogurt (M=1.04±0.24s) and biscuits (M=1.43±0.35s), while water swallows were markedly shorter (M=0.79±0.28s).

In addition to texture effects, inter-subject variability was quantified using the variance components of the mixed-effects model with a random intercept per participant. The estimated between-subject variance (0.0375) reflects systematic differences in average swallowing duration across individuals, whereas the within-subject residual variance (0.1229) captures intra-individual variability and unexplained fluctuations. These components correspond to an intraclass correlation coefficient (ICC) of 0.234, indicating that approximately 23% of total swallowing-duration variability is attributable to inter-individual differences.

Substantial intra-individual variability was observed as shown in Figure 9b. Even within the same participant, swallowing durations ranged from less than 0.5 s to more than 3 s.

### 3.2. Binary Classification Results

In this application, minimizing both false negatives (FNs) and false positives (FPs) is equally important. Therefore, the F1-score is used as the main evaluation metric, as it provides a balance between precision and recall. The model was evaluated using 5-fold cross-validation. Mean performance, summarized in Table 2, was strong and consistent across folds (Accuracy 0.874±0.013, F1-score 0.875±0.013). The classifier demonstrated excellent discriminative ability, achieving an area under the receiver operating characteristic curve (AUROC) of 0.942±0.010 and an area under the precision-recall curve (AUPRC) of 0.931±0.014. Small fold-to-fold variations were observed. The lowest AUROC/AUPRC (Fold 5) can be attributed to comparatively lower segment counts and higher intra-subject variability in swallowing duration in the corresponding participant split, reducing training diversity.

**Figure 10:**
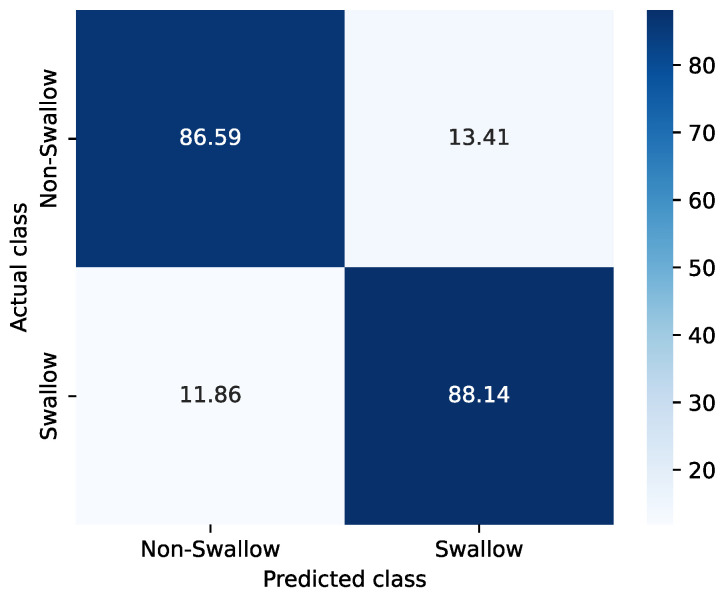
Average confusion matrix across five folds.

The confusion matrix (Figure 10) shows balanced behavior across classes. Averaged over folds, the classifier correctly identified 86.6% of non-swallows, with a corresponding false-positive rate of 13.4%. For swallows, the average true-positive rate was 88.1%, with 11.9% missed swallows (FNs).

To assess the influence of acoustic conditions, performance was also evaluated separately for each environment (quiet, babble, and factory noise), as summarized in Table 3. Overall, the classifier demonstrated stable performance across noise conditions. Performance slightly decreased in the babble noise condition (AUROC 0.930±0.01, F1-score 0.850±0.014), which may be attributed to the spectral overlap between human babble noise and swallowing sounds. The limited impact of background noise on classification performance may be explained by the acoustic seal created by the ear tips, which passively attenuates external noise while preserving internally generated physiological signals such as swallowing sounds.

## 4. Discussion and Limitations

Post-classification analyses showed consistent performance (F1 = 0.875) in all folds, indicating that in-ear audio carries sufficient information for swallow/non-swallow discrimination between individuals.

Across folds, the majority of false positives came from saliva mix segments that were labeled as non-swallow but are acoustically similar to saliva swallows, especially to the oral preparatory phase, when the tongue contracts to propel the bolus against the soft palate.

FNs were dominated by saliva swallows (50.5%), followed by water (19.1%), yogurt (18.9%) and biscuits (11.4%). FNs were most often aligned with atypical or prolonged swallowing patterns. As detailed in Section 2.1.2, participants were asked to swallow their saliva repeatedly over a 30 s window. This repetitive task progressively dries the oral cavity, reducing the available saliva. In practice, participants produced roughly five swallows within that window, the fourth and fifth swallows tended to deviate from typical patterns showing longer durations and more fragmented acoustics because repeated, effortful dry swallowing is harder to execute. These atypical dynamics diverge from the normal swallow signature and, therefore, were more frequently missed by the model. Such task-induced modulation of swallowing timing and dynamics in healthy individuals has been previously reported, including differences between cued and non-cued swallowing conditions [36].

Despite the careful design and implementation of our data acquisition protocol, several limitations must be recognized. Although the dataset includes multimodal recordings from a chest belt (ECG, respiration, and acceleration), these signals were not always available with optimal quality. In particular, ECG recordings were occasionally missing or degraded.

Another limitation is the use of fixed 4 s segments for classification, which may include extended low-activity portions in non-swallow samples and reduce temporal precision. Future work will compare shorter and sliding-window segmentations.

Label validity and reproducibility is also a limitation. Annotation timing is bounded by the 30 fps ultrasound resolution, and labels were generated by a single rater with partial expert review. In the absence of reliability assessment (Cohen’s kappa) and a label validation study, residual label uncertainty cannot be excluded.

It is also important to note that the dataset consists exclusively of healthy young adults aged 20 to 29 years. As a result, it may not adequately represent populations with different physiological characteristics or medical conditions, such as older adults or individuals with swallowing problems. The primary objective of this study was to establish proof of concept in a healthy population. Future work will extend the dataset to elderly individuals and patients with swallowing disorders, such as dysphagia or Parkinson’s disease, to evaluate clinical generalizability.

The classification experiment was designed as a baseline proof of concept. We intentionally used a lightweight FCNN on top of frozen YAMNet embeddings and did not attempt to optimize performance with more complex architectures (convolutional recurrent neural networks (CRNNs), Transformers). Preliminary experiments with from-scratch models (CNNs trained on log-Mel spectrograms and per-channel energy normalization (PCEN) representations) showed lower cross-subject generalization than the transfer-learning approach. Although ultrasound and chest-belt signals were collected, they were not used as auxiliary classifier inputs in this study because our objective was to evaluate a practical, non-invasive swallow detection approach based only on in-ear audio. Future work will investigate multimodal learning and fusion, and more complex models.

## 5. Conclusions

In this work, we present the first comprehensive multimodal dataset dedicated to swallowing and related non-verbal events, recorded using IEM and synchronized physiological signals. It addresses the current lack of publicly available resources for training and validating swallowing detection algorithms. Due to the wide range of tasks performed in both quiet and noisy environments, this dataset is well suited for developing robust and real-world systems across various research and clinical projects. As an initial proof of concept, an FCNN trained on YAMNet+ZCR embeddings achieved a mean F1-score of 0.875, supporting the feasibility of in-ear audio-based swallow detection and motivating future multimodal and clinical validation.

## Figures and Tables

**Table 1:** Comparison of Representative In-Ear and Ear-Worn Datasets.

Dataset	Subjects	Modalities	Noise Diversity
SpEAR	25	IEM + OEM + Ref mic	Quiet + noisy
VibraVox	188	IEM + Bone conduction mic + Laryngophone	Quiet + noisy
iBad	25	IEM + OEM + ECG + Resp.	Quiet + noisy
RespEar	18	IEM + Resp. ref.	Sedentary + active
OESense	31	IEM	Head movement + music
Proposed Dataset	34	IEM + OEM + ECG + Resp. + IMU	Quiet + noisy

IEM: in-ear microphone, OEM: outer-ear microphone, ECG: electrocardiogram, Resp.: respiration, IMU: inertial measurement unit.

**Table 2:** Five-fold cross-validation metrics. Best values per metric are in bold.

Fold	Accuracy	F1	AUROC	AUPRC
Fold 1	0.880	0.878	0.949	0.943
Fold 2	**0.889**	**0.895**	**0.949**	**0.944**
Fold 3	0.873	0.873	0.943	0.932
Fold 4	0.872	0.864	0.945	0.927
Fold 5	0.854	0.864	0.924	0.909
Mean ± SD	**0.874 ± 0.013**	**0.875 ± 0.013**	**0.942 ± 0.010**	**0.931 ± 0.014**

**Table 3:** AUROC and F1-score across noise conditions.

	Quiet		Babble		Factory	
	AUROC	F1	AUROC	F1	AUROC	F1
Mean	0.952	0.890	0.930	0.850	0.944	0.884
SD	±0.010	±0.013	±0.010	±0.014	±0.011	±0.011

## Data Availability

The dataset includes synchronized in-ear and outer-ear audio recordings, physiological signals (ECG, respiration, and accelerometry), ultrasound imaging of tongue motion, and expert-validated annotations. Due to the presence of identifiable speech data, the dataset is not publicly released and is available from the corresponding author upon reasonable request, subject to ethical approval and data-sharing agreements.
